# Stretchable and self-healable spoof plasmonic meta-waveguide for wearable wireless communication system

**DOI:** 10.1038/s41377-022-01005-1

**Published:** 2022-10-25

**Authors:** Bu-Yun Yu, De-Wei Yue, Ke-Xin Hou, Lu Ju, Hao Chen, Cong Ding, Zhen-Guo Liu, Yun-Qian Dai, Hari Krishna Bisoyi, Ying-Shi Guan, Wei-Bing Lu, Cheng-Hui Li, Quan Li

**Affiliations:** 1 State Key Laboratory of Millimeter Waves, School of Information Science and Engineering, Southeast University, Nanjing, 210096 China; 2 Center for Flexible RF Technology, Frontiers Science Center for Mobile Information Communication and Security, Southeast University, Nanjing, 210096 China; 3 Purple Mountain Laboratories, Nanjing, 211111 China; 4 State Key Laboratory of Coordination Chemistry, School of Chemistry and Chemical Engineering, Nanjing University, Nanjing, 210023 China; 5 Institute of Advanced Materials and School of Chemistry and Chemical Engineering, Southeast University, Nanjing, 211189 China; 6 Advanced Materials and Liquid Crystal Institute and Chemical Physics Interdisciplinary Program, Kent State University, Kent, OH 44242 USA

**Keywords:** Metamaterials, Microwave photonics

## Abstract

Microwave transmission lines in wearable systems are easily damaged after frequent mechanical deformation, posing a severe threat to wireless communication. Here, we report a new strategy to achieve stretchable microwave transmission lines with superior reliability and durability by integrating a self-healable elastomer with serpentine-geometry plasmonic meta-waveguide to support the spoof surface plasmon polariton (SSPP). After mechanical damage, the self-healable elastomer can autonomously repair itself to maintain the electromagnetic performance and mechanical strength. Meanwhile, the specially designed SSPP structure exhibits excellent stability and damage resistance. Even if the self-healing process has not been completed or the eventual repair effect is not ideal, the spoof plasmonic meta-waveguide can still maintain reliable performance. Self-healing material enhances strength and durability, while the SSPP improves stability and gives more tolerance to the self-healing process. Our design coordinates the structural design with material synthesis to maximize the advantages of the SSPP and self-healing material, significantly improving the reliability and durability of stretchable microwave transmission lines. We also perform communication quality experiments to demonstrate the potential of the proposed meta-waveguide as interconnects in future body area network systems.

## Abstract (web summary)

A novel stretchable spoof plasmonic meta-waveguide with excellent stability, superior durability, and self-healing ability is achieved, which could ensure reliable microwave transmission in future flexible wireless communication systems.

## Graphical abstract

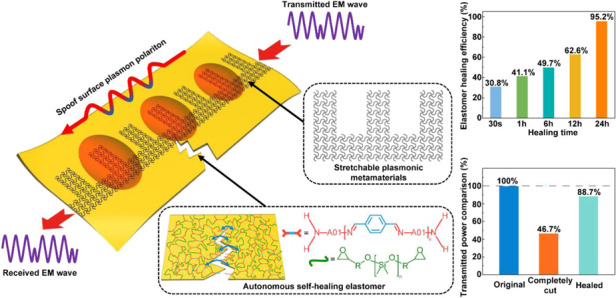

## Introduction

As an indispensable part of wireless communication systems, flexible and stretchable microwave devices have great application value in the field of wearable systems, including electronic skins and biomedical equipments^1–5^. In recent years, various types of stretchable microwave devices have been extensively studied^6,7^. However, stretchable microwave devices now face a severe challenge: elastic substrates and metallic structures are easily damaged after several deformations, resulting in severe performance degradation. The reason for this phenomenon is that the electromagnetic properties of microwave devices are sensitive to structural changes. Especially for microwave transmission lines, slight structural damage could lead to a severe decline in transmission efficiency^8–11^. During long-term use, frequent bending, twisting and stretching are inevitable in wearable systems^12^; thus, structural damage in microwave transmission lines is one of the greatest threats to the stability of wireless communication. At present, conventional elastic substrates and metallic structural design cannot solve this difficult problem.

The application of self-healing materials is a promising way to improve the reliability and durability of stretchable microwave transmission lines. Self-healable polymer materials can repair themselves after mechanical damage and overcome the problem of irreversible performance degradation in damaged devices fabricated by traditional materials^13,14^. However, to obtain a self-healable microwave transmission line with high performance and good reliability, many technical obstacles still need to be overcome. Most self-healing materials cannot simultaneously achieve the characteristics of autonomous self-healing ability, good mechanical strength, and appropriate dielectric properties, which are essential for the performance and reliability of microwave devices^15–20^. For conductive structure design, self-healable conducting composites are still difficult to apply in microwave transmission lines due to their relatively low electrical conductivity (approximately two orders of magnitude lower than common metals) and poor stability (electrical conductivity will decrease significantly when the conductive composite is stretched)^21–25^. Mechanical stretchable metallic structures offer good electrical conductivity and reliability, but the integration between metallic structures and the elastic substrate is complicated^6,7,26^. Moreover, the mechanical stretchable metallic structure cannot repair itself after being damaged due to the lack of self-healing ability. In addition, the self-healing process generally takes at least several hours at room temperature; thus, it is difficult for devices to maintain reliable transmission when the self-healing process has not been completed or the eventual healing effect is not ideal. It is difficult to simultaneously overcome these bottlenecks by innovating structural design or improving material synthesis alone.

**Fig. 1:**
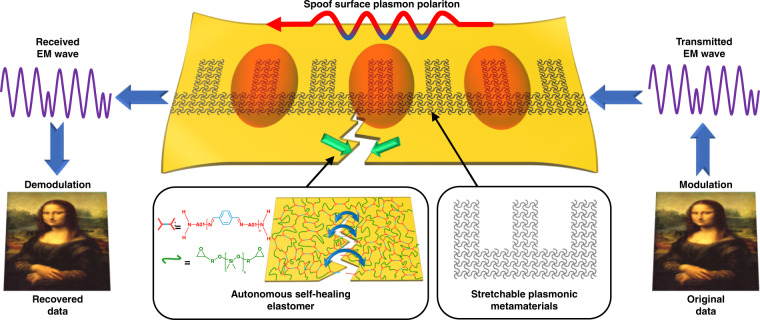
Conceptual illustration of the stretchable and self-healable spoof plasmonic meta-waveguide. Self-healing material enhances structural strength and durability, while the SSPP structure improves reliability and gives more tolerance to the self-healing process

In this article, we present a new strategy to fabricate stretchable microwave transmission lines by integrating a self-healable elastomer with specially designed plasmonic metamaterials. An illustration of the proposed strategy is depicted in Fig. 1. Based on epoxy-polyimine covalent adaptive network (CAN), we designed and synthesized a self-healable elastomer as the microwave substrate. The optimized epoxy-polyimine CAN possesses excellent mechanical properties, autonomous self-healing healing ability and appropriate dielectric characteristics. After autonomous healing for 24 h at room temperature, the healing efficiency reached 95.3%. In terms of conductive structure design, we innovatively introduced the serpentine geometry strategy into plasmonic metamaterials to support the propagation of the spoof surface plasmon polariton (SSPP). Owing to the unique field distributions of SSPPs^27–32^, like natural SPPs in the optical regime, the fabricated spoof plasmonic meta-waveguide exhibits stable performance under the conditions of extreme deformation and partial damage. Even if the self-healing process has not been completed or the eventual repair effect is not ideal, the meta-waveguide can still maintain reliable performance. The self-healing material enhances strength and durability, while the SSPP improves stability and provides more tolerance for the self-healing process. The proposed strategy closely coordinates the structural design with material synthesis to maximize the advantages of the SSPP and self-healing material, promoting the reliability of microwave transmission lines to a higher level. In addition, owing to the strong interaction between the epoxy group and metal surface, the metallic structure, and elastic substrate can tightly bond together without the help of any adhesive or equipment, which greatly facilitates the fabrication process. The strong stability, autonomous self­healing characteristics, excellent damage resistance and low manufacturing cost make the proposed stretchable spoof plasmonic meta-waveguide a potential candidate in the field of electronic skins, wearable medical devices, and other flexible wireless communication systems.

## Results

**Fig. 2:**
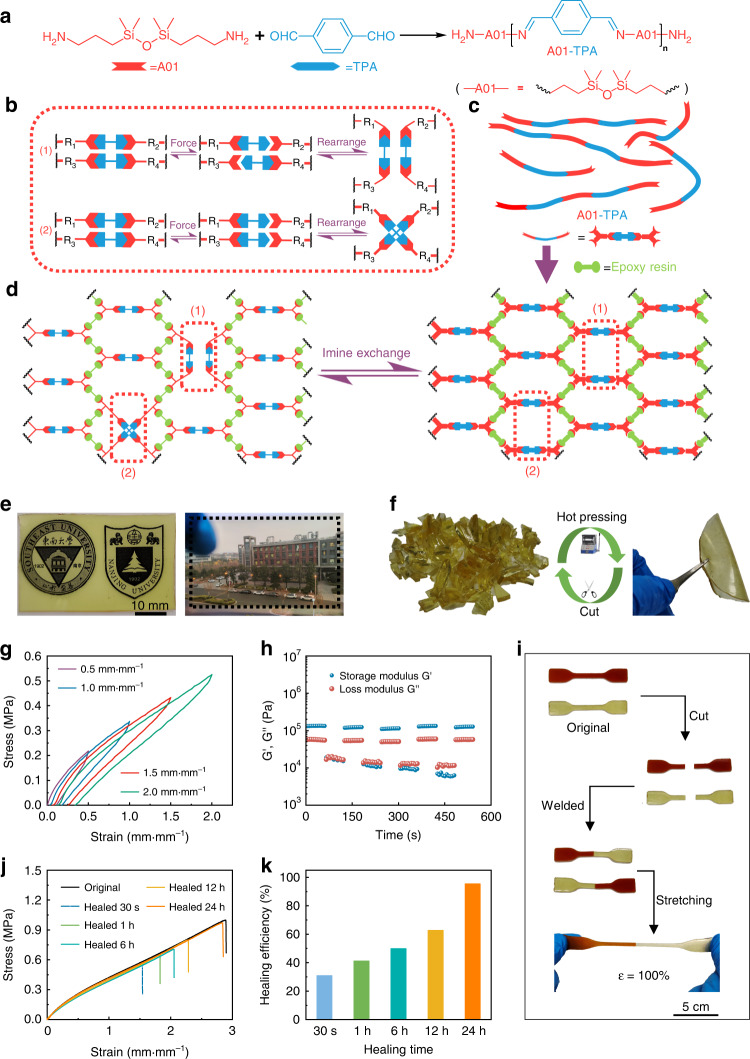
The fabrication of ATPA-EP and its mechanical and self-healing performance. **a** Synthesis route of A01-TPA and its structure. **b** Associative exchange mechanisms of epoxy-polyimine CAN. **c** Simplified schematic of A01-TPA. **d** The crosslinking dynamic ATPA-EP network. **e** The as-prepared transparent ATPA-EP film and **f** its reprocessability. **g** Cyclic loading–unloading curves of ATPA-EP from 50 to 200% strain without relaxing time. **h** Continuous step strain measurements of ATPA-EP at 25 °C. **i** Photographs depicting the macroscopic cutting–welding–stretching procedure of two ATPA-EP samples with different colors at room temperature. **j** Typical stress–strain curves for the cut ATPA-EP films that were healed under different time scales. **k** The self-healing efficiencies of ATPA-EP films under different time scales

An effective way to achieve self-healing ability is incorporating dynamic imine bonds into polymers because of the diversity of reactants and the low activation energy for bond exchange^33,34^. However, a design that completely adopts imine bonds as the dynamic cross-linking network has certain drawbacks, such as low material strength and uncontrollable fabrication^17,18^, which can significantly affect the stability of microwave devices. CANs that combine dynamic covalent bonds with crosslinked network structures are currently at the forefront of research. Their internal topology network structure can be rearranged, which enables materials to be self-healing and reprocessable without loss of macroscopic properties^19,35^. An epoxy-polyimine CAN is an optimized strategy since epoxy resin possesses excellent mechanical properties. Moreover, the abundant hydroxyl groups in cured epoxy resin exhibit good adhesion to metallic structures, which facilitates the integration of components and improves device stability. The preparation of epoxy resin is simple, and the curing process is solvent-free. Most importantly, the epoxy resin has an appropriate dielectric constant and dielectric loss, which are essential for microwave devices. The synthesis route of our self-healing elastomer is presented in Figs. 2a and S1. 1,3-Bis(3-aminopropyl)-1,1,3,3-tetramethyldisiloxane (A01) and terephthalaldehyde (TPA) were reacted in a certain ratio, obtaining amino-terminated linear polyimine with a number-average molecular weight of approximately 15000 g mol^−1^ (denoted A01-TPA, Figs. 2c and S2). After postcuring with silicone-based epoxy resin, a crosslinking polymer with a dynamic imine CAN was prepared (denoted ATPA-EP). Figure 2b, d illustrate the associative exchange mechanisms of the epoxy-polyimine CAN. The original imine bond is broken and associates with another bond without any loss in network integrity^17^, endowing the epoxy-polyimine CAN with reprocessability and self-healing ability. Figure 2e depicts the reprocessability of the polymers. A transparent polymer film was first cut into small fragments and then hot-pressed at 50 °C under a pressure of 20 kPa for 5 min to obtain a new polymer film.

The details of the general characterization of A01-TPA and ATPA-EP can be found in Figs. S3–S7. There is no obvious aggregation according to the XRD patterns, and the measured glass transition point (Tg) of ATPA-EP is 5.08 °C. The thermal stability of ATPA-EP was enhanced after crosslinking. As revealed by the heating-cooling rheological temperature sweeps (Fig. S8), the temperature-dependent modulus of ATPA-EP during cooling is nearly the same as that during heating, indicating good thermal reversibility and stability of the elastic polymer network. In addition, multiple frequency small-amplitude oscillatory sweep (mf-SAOS) experiments were conducted under different temperatures to investigate the dynamic property of the polymer. The data at selected temperatures are shown in Fig. S9a. Both *G*′ and *G*′′ decrease monotonically with temperature at high angular frequencies, but at relatively low frequencies, *G*′′ drops first and then rises with decreasing temperature. According to the time–temperature superposition (TTS) principle, this trend is consistent with the master curve at a reference temperature of *T*_ref_ = 20 °C (Fig. S9b), which could be ascribed to the dynamic reversibility of ATPA-EP. In addition, the experimental data of αT depicted in Fig. S10 cannot perfectly match the Arrhenius equation (Eq. 1) over the full temperature range, but the equation still takes effect over a wide temperature window. The calculated activation energy *E*_*a*_ is as low as 54.96 kJ mol^−1^, suggesting the rapid dynamic exchange of ATPA-EP, which results in room-temperature self-healing properties.

1 $$\ln a_T = \frac{{1000E_a}}{{RT}} - \ln A$$

The electromagnetic characteristic of the ATPA-EP is measured by a split post dielectric resonator. The dielectric constant of ATPA-EP is approximately 2.63, and the dielectric loss tangent is approximately 0.02 (measured at 5.1 GHz). The appropriate electromagnetic characteristics make ATPA-EP a suitable candidate for acting as a microwave substrate.

To investigate the mechanical properties of ATPA-EP, the cyclic loading–unloading curves under successive strain are shown in Fig. 2g. There is a hysteresis that verifies an efficient energy dissipation from dynamic imine bonds. To evaluate the self-healing properties, continuous oscillatory step strain experiments were conducted. As illustrated in Fig. 2h, small and large strains were carried out alternatively. In the first 60 s, the initial *G*′ is larger than *G*′′ at a small strain (0.1%), and the elastic-dominant viscoelasticity verifies the integrity of the crosslinked network. In the next 60 s, *G*′′ is greater than *G*′, and *G*′ decreases by almost 1 order of magnitude when a large strain is applied (300%), indicating a partially broken crosslinked network. After the strain amplitude returned to a small value in the next 60 s, *G*′ and *G*′′ recovered to their original state without hysteresis, which verifies the excellent self-healing performance of ATPA-EP. This ability can be ascribed to the fast associative exchange mechanisms of epoxy-polyimine CAN. Figure S11 shows the optical microscope images that directly exhibit the scratching–healing process. The scratches on the film gradually disappeared after healing for 24 h at room temperature. Furthermore, as Fig. 2i depicts, an original sample and a red-colored sample were cut into two separated parts using a razor blade, and then half of each sample was welded with half of the other sample. After self-healing at room temperature for 24 h, the resulting dichromatic sample was able to stretch to twice its original length. Figure 2j represents typical stress–strain curves for the cut ATPA-EP films that were healed under the different time scales. The original sample possesses excellent mechanical properties with an elongation at break of 274 ± 15% and a Young’s modulus of 0.71 ± 0.09 MPa. To quantitatively analyze the self-healing properties of materials, the healing efficiency (*η*) is defined as the ratio of the fracture energy of the healed sample to the original sample (Eq. 2).

2 $$\eta = \frac{{E_{\rm{healed}}}}{{E_{\rm{original}}}} = \frac{{S_{\rm{healed}}}}{{S_{\rm{original}}}}$$

The fracture energy of the sample is equal to the integrated area under the stretch curve. As shown in Fig. 2k, the ATPA-EP film could be quickly healed after 30 s with *η* = 30.80%. After healing for 24 h, the healing efficiency η reached 95.29%.

**Fig. 3:**
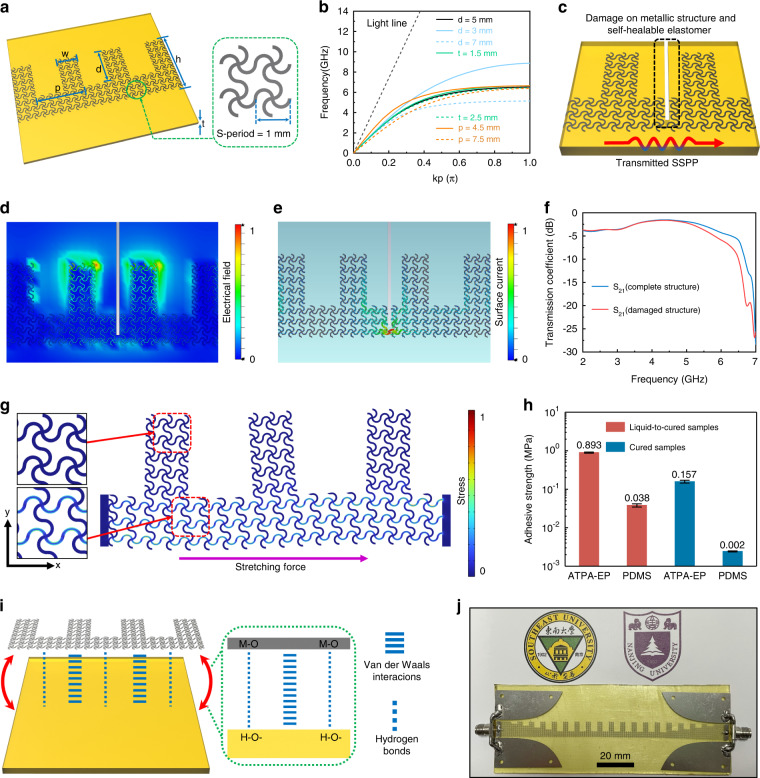
Design and fabrication of the self-healable and stretchable spoof plasmonic meta-waveguide. **a** Serpentine-type plasmonic metamaterial unit. **b** Simulated dispersion curves of the plasmonic metamaterial unit. **c** Illustration of the damaged structure. **d** Simulated electrical field and **e** surface current distribution of the damaged meta-waveguide. **f** Transmission coefficient comparison between damaged and complete meta-waveguides. **g** Mechanical strain simulation of the proposed stretchable meta-waveguide. **h** Measured adhesive strength of ATPA-EP and PDMS on the steel surface. **i** Illustration of the integration between the epoxy polyimide elastomer (ATPA-EP) and metallic structure. **j** Photograph of the fabricated spoof plasmonic meta-waveguide

In terms of metallic structural design, we introduced the serpentine geometry design strategy into plasmonic metamaterials to support the propagation of SSPPs for the first time. Owing to the excellent electrical conductivity of the serpentine-type metallic structure, this design strategy is especially appropriate for fabricating microwave devices that are very sensitive to metal loss, such as microwave transmission lines. To easily convert the conventional structure into a serpentine-type conductor, we chose the single-side corrugated groove to act as the plasmonic metamaterial unit (Fig. 3a). According to the previous literatures^36^, the dispersion relation of the TM-polarized waves propagating along one-side groove structures can be obtained from the following equation (Eq. 3).

3 $$k_x = k_0\sqrt {1 + \frac{{(p - w)^2}}{{p^2}}{\rm{tan}}^2(k_0d)}$$

The *k*_0_ *=* *ω/c* represents the wave number in free space, and *c* is the velocity of light. When 0 < *k*_0_*d* < π/2, the *k*_*x*_ will be larger than *k*_0_, which indicates the slow wave characteristic of the SSPP. It can be observed that the cutoff frequency of the periodic structure is sensitive to the depth of the groove. The simulated dispersion curves are in good agreement with the theoretical analysis (Fig. 3b). We chose a depth *d* = 5 mm (*w* = 2.6 mm, *h* = 8.1 mm, *t* = 2 mm, *p* = 6 mm) to cover most commonly used wireless communication bands. To match the depth of the groove unit, the period of the serpentine-type unit (S-period) is selected to be 1 mm, and the width of metallic line is 100 μm. To illustrate the damage resistance ability of the serpentine-type meta-waveguide, we simulated the electromagnetic performance and field distributions of the damaged serpentine-type meta-waveguide in CST microwave studio, which is shown in Fig. 3c–f. From the simulated S-parameters and field distributions, it can be observed that the transmitted SSPPs remain stable even if there is a huge damage on the metallic structure and elastic substrate. The electrical fields are mainly located on the groove structures, while surface currents are mainly distributed on the connecting structures between the grooves. As long as the connecting part is not completely broken, it can still provide complete surface current path for the whole meta-waveguide, and the transmission performance will remain stable. Moreover, we simulated the mechanical properties of the proposed structure, which is shown in Fig. 3g. It can be observed that the serpentine-type meta-waveguide has no obvious stress and deformation in the *y*-axis direction. Because the electromagnetic characteristic of the spoof plasmonic meta-waveguide is most sensitive to the depth of the groove, the proposed serpentine-type meta-waveguide will maintain stable performance while it is stretched and deformed. Even if the asymptotic frequency characteristic of the spoof plasmonic meta-waveguide changes slightly, the meta-waveguide can still maintain reliable transmission in the lower frequency band. The meta-waveguide structure is first manufactured from a 100 μm metal film (304 steel or copper) and transferred onto the epoxy polyimide elastomer. The –OH functional group in the epoxy polyimide elastomer can form hydrogen bonds with the metal surface; thus, the metallic structure and elastomer can tightly bond together without the use of any adhesive or equipment (Fig. 3i). In Fig. 3h, it can be observed that the measured adhesive strength of the liquid-to-cured ATPA-EP and cured ATPA-EP on the metallic surface is 0.893 MPa and 0.157 MPa, respectively, which is much higher than that of PDMS (0.03 and 0.002 MPa, respectively). Owing to the simple integration process and low manufacturing cost, the proposed spoof plasmonic meta-waveguide has the potential for mass production. The fabricated self-healable and stretchable spoof plasmonic meta-waveguide is shown in Fig. 3j. To facilitate the connection between the prototype and vector network analyzer, we fabricated gradient transition structures at both ends of the meta-waveguide to achieve mode conversion between guided waves on a coplanar waveguide and SSPPs on plasmonic metamaterials^36^. The detailed geometric parameters are shown in Fig. S13.

**Fig. 4:**
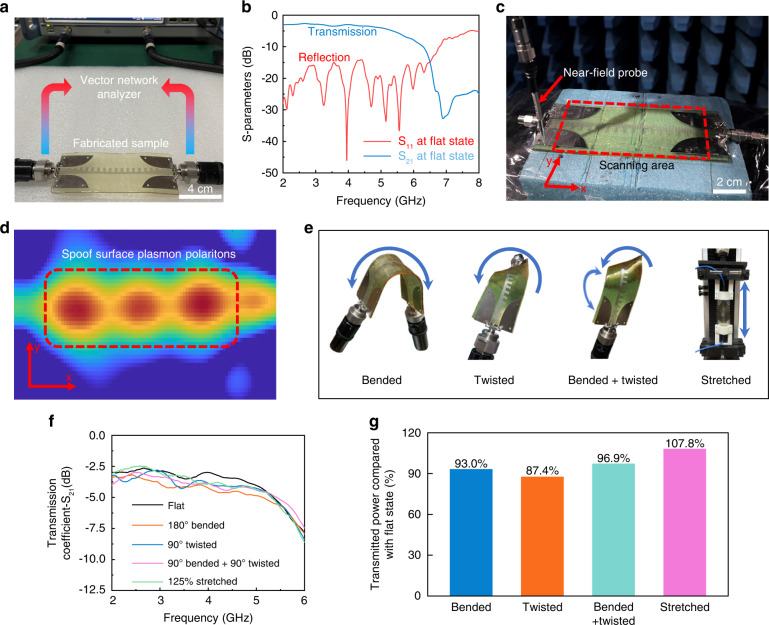
Measurement of the undamaged spoof plasmonic meta-waveguide. **a** Experimental photograph of the meta-waveguide in the flat state. **b** Measured S-parameters of the flat sample. **c** Near-field distribution measurement of the meta-waveguide. **d** Measured near-field distributions of the meta-waveguide. **e** Experimental photograph of the meta-waveguide with different deformed state. **f** Measured transmission coefficients and **g** power comparison of the deformed samples

To validate the effectiveness of the design strategy, we experimentally measured the electromagnetic properties of the proposed spoof plasmonic meta-waveguide under the conditions of being extremely deformed, mechanically damaged, and autonomously healed. First, we measured the S-parameters of the meta-waveguide at the condition of the flat state, which is shown in Fig. 4a. S_21_ is the transmission coefficient that represents the electromagnetic power transmission, and S_11_ indicates the intensity of the power reflection. From the measured results (Fig. 4b), it can be observed that the meta-waveguide shows high transmission efficiency from 2 to 5 GHz. Compared with the meta-waveguide fabricated by conventional metal (Fig. S14), the proposed stretchable meta-waveguide exhibits the same transmission efficiency, which demonstrates the excellent electrical performance of the serpentine-type metallic structure. The near-field distributions (*E*_*z*_ components) of the self-healable and stretchable spoof plasmonic meta-waveguide were obtained in an anechoic chamber (Fig. 4c). From the measured results (Fig. 4d), it can be clearly observed that the SSPPs are excited on serpentine-geometry plasmonic metamaterials. Then we verified the electromagnetic performance of the proposed meta-waveguide under several types of deformation, which is shown in Fig. 4e. The fabricated sample retains stable performance under the conditions of 180° bending, 90° twisting, 90° bending and 90° twisting simultaneously, and 125% stretching (Figs. 4f, g and S15). Owing to the unique field distributions of SSPPs, the proposed spoof plasmonic meta-waveguide exhibits excellent stability under extreme and complex deformation. In practical use, wearable electronic systems should inevitably withstand repeated and prolonged deformation, and the proposed meta-waveguide can guarantee the reliability of microwave transmission in complex working situations.

**Fig. 5:**
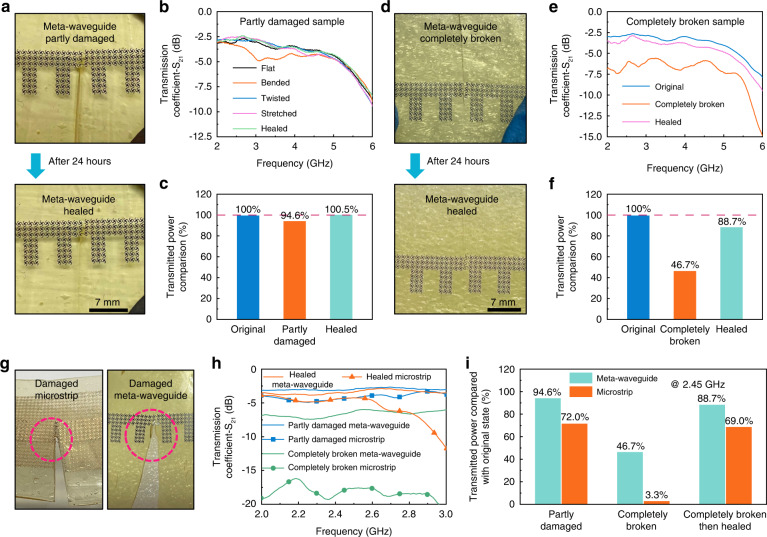
Damage resistance and self-healing performance of the spoof plasmonic meta-waveguide sample. **a** Photograph of the partly damaged and healed sample. **b** Measured transmission coefficients of the damaged sample. **c** Transmitted power comparison between the damaged sample and healed sample. **d** Photograph of the completely broken and healed sample. **e** Measured transmission coefficients of the completely broken sample. **f** Transmitted power comparison between the broken sample and healed sample. **g** Damage resistance experiment between the proposed meta-waveguide and microstrip line. **h**, **i** Transmission comparison between the proposed meta-waveguide and the microstrip line

After repeated deformations, wearable systems are likely to suffer structural damage. However, as the crucial component of wireless communication, the electromagnetic performance of microwave transmission lines is sensitive to structural changes. To overcome this difficult problem, we coordinate the plasmonic metamaterial with self-healable material to enhance the reliability and durability of microwave waveguides. We performed several damage resistance and self-healing experiments on the proposed meta-waveguide to verify the effectiveness of our design strategy. First, we cut part of the self-healable elastomer and metallic structure of the fabricated sample, and measured the electromagnetic characteristics of the partly damaged prototype, which is shown in Fig. 5a. The detailed measurement photos and S-parameters are depicted in Fig. S16. The structural deformation worsens when the damaged meta-waveguide is bent, twisted, and stretched. From Fig. S16a, it can be observed that the elastic substrate is almost completely torn apart under stretching and twisting. In this case, the damaged meta-waveguide can still maintain stable performance even with severe structural deformation (Fig. 5b, c). These experimental results illustrate that the proposed structure exhibits excellent damage resistance, which is critical for the stability of microwave transmission.

After the above measurements, we exposed the partly damaged meta-waveguide in the air for 24 h at room temperature. Within 24 h, the damaged elastic substrate autonomously recovered without external pressure. The self-healed prototype is shown in Fig. 5a, and it can be observed that the wound on the substrate basically healed. We measured the self-healed meta-waveguide, and there is almost no difference between the performance of the original and healed prototype (Fig. 5c). The healed meta-waveguide’s physical strength and electromagnetic performance are comparable to the original states. It is noteworthy that there is still an uneven scar around the metallic structure. The substrate near the metallic structure was severely deformed during the damage experiment, so the healing effect in this area is not particularly good. However, the uneven scar on the elastomer has no influence on the transmission performance, which demonstrates the adaptability of the SSPP structure. Due to severe damage or deformation, the self-healable elastomer may not fully recover to the original state after the self-healing process, and the introduction of the SSPP overcomes this problem. From the simulated results of the dispersion curves, we can conclude that the electromagnetic characteristic of the plasmonic metamaterial is not sensitive to the thickness of the elastic substrate. The structural deformation may cause a slight frequency shift, but it has no obvious influence on the transmission efficiency at lower frequencies. Therefore, even if the elastomer cannot be completely restored to its original condition, the proposed meta-waveguide can still maintain its original performance. The adaptability charateristic of SSPPs is crucial, as it gives more tolerance to the self-healing process and enhances stability. For conventional transmission lines (such as microstrip lines), if the signal line conductor is completely broken, the data transmission will completely fail. To imitate this worst-case scenario, we completely cut the metallic structure and measured its performance (Figs. 5d, e and S17). Since there is no conductive path between the two separated conductors, the transmission efficiency of the spoof plasmonic meta-waveguide is affected to a certain degree. However, even if the metallic structure is completely cut and slightly separated, the transmission efficiency of the severe damaged meta-waveguide can still reach approximately half of the original sample (46.7% at 2.45 GHz), which is shown in Fig. 5f. At the notches of the conductors, a part of the electromagnetic energy can be coupled to the other side by near-field excitation. This unique characteristic of SSPPs allows the meta-waveguide to retain a part of the transmission efficiency under extreme conditions. We brought the separated structures into contact and placed the sample in the air for 24 h. When the substrate is healed and the conductive connections are re-established, the transmission efficiency of the healed meta-waveguide can be restored to its original performance. We performed comparison experiments between the proposed meta-waveguide and microstrip line (Fig. 5g–i). It can be observed that the reliability of the proposed spoof plasmonic meta-waveguide is significantly superior to the microstrip line. In particular, when the signal line is completely broken, the transmitted power of the proposed meta-waveguide is much higher than that of the microstrip line. The SSPPs provides more tolerance to the self-healing process, while the self-healing material enhances the strength and durability of the spoof plasmonic meta-waveguide. The proposed design strategy maximizes the advantages of SSPPs and self-healing materials, promoting the reliability of microwave transmission lines to a higher level.

**Fig. 6:**
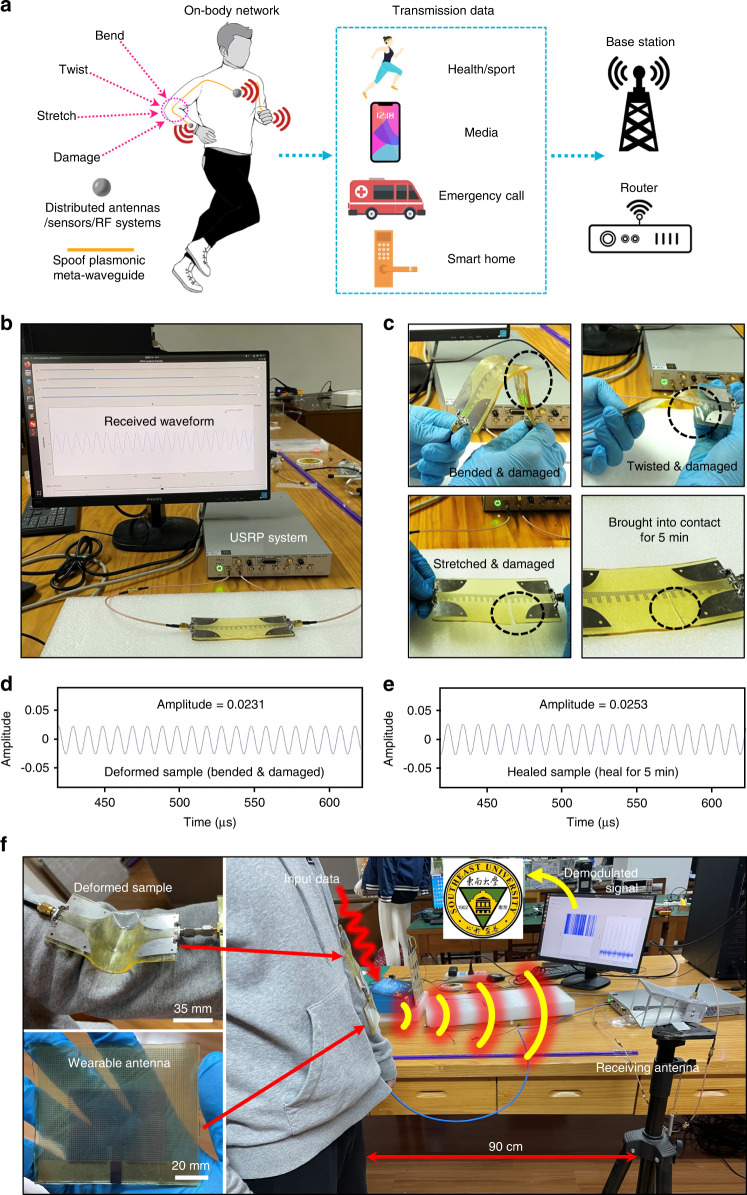
Communication performance of the spoof plasmonic meta-waveguide. **a** Conceptual illustration of the body area network based on the spoof plasmonic meta-waveguide. **b** Measurement setup of the waveform transmission experiment. **c** Photos of the damaged and deformed meta-waveguide. **d** Received waveform of the bend and damaged sample. **e** Received waveform of the healed sample. **f** On-body wireless communication experiment of the spoof plasmonic meta-waveguide with the help of the USRP system and wearable antenna

The excellent stability, autonomous self­healing ability, and superior damage resistance make the proposed spoof plasmonic meta-waveguide promising in future body area network systems, which is depicted in Fig. 6a. The proposed meta-waveguide could be utilized to interconnect antennas, sensors and RF systems distributed on various parts of the human body. To experimentally investigate the influence of deformation, mechanical damage, and the self-healing process on the communication performance, we used a universal software radio peripheral (USRP) system to verify the actual communication performance of the sample. We first carried out a series of waveform transmission experiments on the proposed meta-waveguide (Fig. 6b). We utilized the USRP system to generate a sine wave with a frequency of 2.45 GHz. The amplitude of the received sine wave is approximately 0.0256 when the prototype is undamaged and flat. We partly cut the prototype and then bent, twisted and stretched the structure. The experimental photos and results are shown in Figs. 6c–e and S18. It can be observed that the received waveform is not distorted after passing through the damaged and deformed meta-waveguide sample. In addition, the amplitude of the received waveform has no significant attenuation compared with the original state. The experimental results demonstrate that the damaged and deformed meta-waveguide can still support reliable radio frequency (RF) signal transmission. After autonomously self-healing for 5 min, the amplitude of the received waveform can be almost restored to its original state. Then, to verify the actual performance of the spoof plasmonic meta-waveguide in a wireless communication system on the human body, we fabricated a wearable antenna based on a serpentine-type patch and self-healing elastomer (the antenna operation frequency is 2.45 GHz). The measurement photographs are shown in Fig. 6f. The meta-waveguide sample was placed on the elbow, and we connected one end of the meta-waveguide to the emitting port of the USRP system and connected the other end to the wearable antenna. A horn antenna is placed approximately 90 cm away from the human body and connected to the receiving port of the USRP system. The modulation scheme of the USRP system was set to binary phase shift keying (BPSK). After the experimental environment was set up, we wirelessly transmitted several pictures through the USRP system. The action of the elbow have no influence on the wireless transmission of the picture. Even if the meta-waveguide is partly damaged, no bit error occurs in the wireless transmission process, and the picture data can be received and demodulated normally. Experimental results verify the strong reliability and damage resistance of the proposed meta-waveguide, which can maintain RF signal transmission in various extreme situations. The proposed spoof plasmonic meta-waveguide provides a potential option for reliably transmitting physiological signals, sports data or media information in future on-body wireless communication systems.

## Discussion

In conclusion, we report a self-healable and stretchable spoof plasmonic meta-waveguide by integrating a self-healable elastomer with serpentine-geometry plasmonic metamaterials. We synthesized a type of epoxy-polyimine CAN called ATPA-EP to act as the stretchable substrate. The optimized epoxy-polyimine CAN has the characteristics of high material strength, autonomous self-healing ability and good dielectric properties, which are crucial for the performance and reliability of microwave devices. In addition, we innovatively introduced the serpentine geometry strategy into plasmonic metamaterials to support SSPPs, which endows the meta-waveguide with excellent stretchability and stability without sacrificing electromagnetic performance. The fabricated spoof plasmonic meta-waveguide exhibits stable electromagnetic performance under extreme and complex deformation. Moreover, owing to the unique physical characteristics of SSPPs, the meta-waveguide can maintain reliable performance when the substrate and metallic structure are damaged. After the autonomous self-healing process, the transmission efficiency of the healed meta-waveguide can be restored to its original performance. Self-healing material enhances structural strength and durability, while SSPPs improve stability and provides more tolerance for the self-healing process. Experimental results validate the effectiveness of our design strategy and verify the great potential of the proposed spoof plasmonic meta-waveguide in the field of wearable wireless communication systems.

## Materials and methods

### Chemicals

Terephthalaldehyde (TPA), 5 A molecular sieves and toluene were purchased from Shanghai Macklin Biochemical Co., Ltd. 1,3-Bis(3-aminopropyl)-1,1,3,3-tetramethyldisiloxane (A01) was purchased from Aladdin Chemical Reagent Co., Ltd. Benzyl glycidyl ether was obtained from Anhui Xinyuan Technology Co., Ltd. Epoxypropoxypropyl terminated polydimethylloxane was purchased from Gelest, Inc. All chemicals and solvents were used without further purification. Ecofelx TM 00–30 was purchased from Smooth-On, Inc.

### Instruments

The 1H NMR spectrum (400 MHz) was measured on a Bruker DRX 400 NMR spectrometer at room temperature in deuterated dimethyl sulfoxide (DMSO) without an internal standard. FT-IR curves were recorded via Nicolet iS10 in a wavenumber range of 4000–400 cm^−1^. Thermal gravimetric analysis (TGA) data were obtained on a NETZSCH STA449F3 instrument from 30 to 600 °C with a heating rate of 10 °C min^−1^ under a dry N_2_ atmosphere. Differential scanning calorimetry (DSC) experiments were performed using a METTLER DSC823e apparatus from −50 to 120 °C with a heating rate of 10 °C min^−1^. X-ray differential (XRD) curves were recorded on a powder X-ray diffractometer (Bruker D8 ADVANCE) between 5° and 50° at a scanning rate of 5 K min^−1^. Gel permeation chromatography (GPC) measurements were conducted on a Malvern VE2001 GPC module using toluene as the mobile phase.

### Mechanical tests

Unless otherwise noted, the uniaxial tensile tests were performed on an Instron 3343 instrument at 25 °C, and the tensile speed was 50 mm min^−1^. A standard dumbbell sample (GB 1040.2-2006 5B) with an effective length of 12 mm was adopted for each measurement, and three parallel experiments were performed to ensure data reliability. Successive cyclic tensile tests were performed at different strains from 50 to 200%.

### Rheological test

The rheological measurements were carried out on a TA DHR-2 rotational rheometer using a plate-plate geometry with a diameter of 8 mm. Temperature sweeps were recorded between −10 and 120 °C at a heating or cooling speed of 5 K min^−1^. Multiple frequency small-amplitude oscillatory sweep (mf-SAOS) tests are at a strain amplitude of 0.1% under log-sweep mode. The temperature-resolved mf-SAOS experiments were performed at angular frequencies ranging from 1 to 15 rad/s, and the sample was loaded into the preheated rheometer at 120 °C and cooled in 10 K steps until 20 °C. Each step was interrupted long enough to reach equilibrium.

### Self-healing tests

To investigate the self-healing procedure, the pristine film was first cut into two separate parts via a razor blade. Then, they were placed together without applying pressure and healed at room 25 °C. Finally, the tensile stress–strain curves of the healed sample were recorded in the same procedures according to the abovementioned test. Optical microscopy images were taken using a Nikon ECLIPSE E100 microscope with a DLC-300 industrial camera. The oscillation frequency of the continuous step strain measurement was fixed to 1 Hz, and the small and large strain amplitudes were 0.1 and 300%, respectively. Each scan lasted 60 s.

### Synthesis of dynamic imine oligomer as epoxy-curing agent (A01-TPA)

The **A01-TPA** was synthesized according to the previous work with modification^37^. TPA (14.7 g, 0.11 mol) was added to a 500 ml single-neck round flask and dissolve it with 200 ml of toluene, then A01 (50.1 g, 0.202 mol, 1 mol% excess to facilitate imine bond exchange) was added dropwise under stirring. Several 5 A molecular sieves were added to facilitate removal of by-products water. The mixture was heated to 130 °C and refluxed for 6 h under N_2_ atmosphere. After reaction, filtered out molecular sieves and removed toluene by rotary evaporation. A transparent bright orange viscous liquid was obtained after heating for 8 h in a vacuum dryer at 70 °C (59.9 g, yield 99.2%).

### Synthesis of self-healing substrate (ATPA-EP)

Benzyl glycidyl ether (13.2 g, 0.08 mol) and epoxypropoxypropyl terminated polydimethylsiloxane (20.0 g, 0.06 mol) were mixed via DAC 150FV (FlackTek Inc.) speed mixer at 2400 rpm, the obtained epoxy mixture was denoted as EP. Then, EP (20.0 g) and A01-TPA (20.0 g) were thoroughly mixed and degassed by a speed mixer at 2400 rpm. After that, the bubble-free mixture was poured into a PTFE mold with dimensions of 150 mm (*L*) × 100 mm (*W*) × 2 mm (*H*) followed by curing in an air oven at 80 °C for 4 h. A yellow transparent film was obtained after peeling off from the PTFE mold.

### Electromagnetic characteristic measurement of the as-prepared self-healing ATPA-EP substrate

The electromagnetic characteristics of the prepared self-healing film were obtained by a split post dielectric resonator from QWED (model: F-SPDR-5.1). The resonant frequency and *Q* factor of the split post dielectric resonator were measured by a vector network analyzer (model: Rohde & Schwarz ZNB40). We synthesized a 1.5 mm thick film by using a 50 mm (*L*) × 50 mm (*W*) × 1.5 mm (*H*) PTFE mold. The resonant frequency and *Q* factor of the split post dielectric resonator will change when the film sample is inserted into the resonator cavity. From the difference in the resonant frequencies between the empty resonator and the resonator inserted with the film sample, we can calculate the dielectric constant of the material. The dielectric loss of the material is calculated by the difference in the Q factors.

### Adhesion measurement of the as-prepared self-healing ATPA-EP substrate

All adhesive strength data were obtained via lap-shear strength tests on an Instron 3343 instrument at 25 °C at a constant speed of 1 mm min^−1^ (Fig. S12). Each test was carried out at least 3 times. Two pieces of steel sheets with a size of 60 mm (*L*) × 20 mm (*W*) × 2 mm (*H*) were sanded with 400-mesh sandpaper and washed. Liquid-state PDMS and ATPA-EP were coated on the steel, followed by covering with another steel sheet. The coating area is 20 mm × 14 mm. Then, two pieces of steel sheets were fixed and placed into an 80 °C oven for curing for 2 h. To estimate the adhesive strength of cured ATPA-EP and PDMS toward the steel substrate, the cured samples were cut into pieces with dimensions of 20 mm (*L*) × 14 mm (*W*) × 0.2 mm (*H*). Then, the samples were placed between two steel sheets and fixed, followed by heating for 2 h at 80 °C in an oven. The adhesive strength is determined by the following equation:

4 $$\sigma = \frac{{F_{{\rm{max}}}}}{S}$$

where σ is the adhesive strength, *F*_max_ is the maximum load force during the lap-shear strength test, and *S* is the contact area between the sample and steel sheets.

### Electromagnetic performance measurement of the SSPP spoof plasmonic meta-waveguide

All the electromagnetic performance measurements of the spoof plasmonic meta-waveguide were performed by a vector network analyzer (model: Rohde&Schwarz ZNB40). The meta-waveguide sample is connected to the vector network analyzer by flexible cables to obtain the *S*-parameters. A stretch performance test was performed on a tensile tester (STS500 brought from XIAMEN YISHITE INSTRUMENTS CO.), and we customized 3D-printed fasteners to realize fixation between the sample and tensile tester. In the self-healing performance measurement, the damaged sample was placed in the air at room temperature (approximately 20 °C) for 24 h. Then, the healed sample was connected to the vector network analyzer to obtain the electromagnetic performance.

### Communication performance measurement of the spoof plasmonic meta-waveguide

The communication performance experiments on the spoof plasmonic meta-waveguide were conducted using a USRP system (USRP N310 from Wuhan Luowave). In the waveform transmission experiments, the baseband signal is a 100 kHz sine wave, and the sampling rate is 1 MHz. The center frequency of the electromagnetic wave generated by the USRP system is 2.45 GHz, and the output power of the USRP system is 5 dBm. In the wireless communication experiment, the modulation scheme of the USRP system was set to BPSK, and the output power of the USRP system was set to 10 dBm. The realized gain of the receiving horn antenna is 13 dBi at a frequency of 2.45 GHz.

## Supplementary information

Supplementary Information
