# Supplementary material for: Stretchable and self-healable spoof plasmonic meta-waveguide for wearable wireless communication system
Source: Light Sci Appl. 2022 Oct 25;11:307. doi: 10.1038/s41377-022-01005-1 (PMC9592613; doi:10.1038/s41377-022-01005-1)
Supplement: Supplementary file 1 — Supplementary Information [file 41377_2022_1005_MOESM1_ESM.docx]

Supplementary Information for

**Stretchable and self-healable spoof plasmonic meta-waveguide for wearable wireless communication system**

*Bu-Yun Yu, De-Wei Yue, Ke-Xin Hou, Lu Ju, Hao Chen, Cong Ding, Zhen-Guo Liu, Yun-Qian Dai, Hari Krishna Bisoyi, Ying-Shi Guan, Wei-Bing Lu*, Cheng-Hui Li*, and Quan Li**

B.-Y. Yu, L. Ju, H. Chen, C. Ding, Z.-G. Liu, W.-B. Lu

State Key Laboratory of Millimeter Waves, School of Information Science and Engineering, Southeast University, Nanjing 210096, China.

Email: [wblu@seu.edu.cn](mailto:wblu@seu.edu.cn)

B.-Y. Yu, L. Ju, H. Chen, C. Ding, Z.-G. Liu, W.-B. Lu

Center for Flexible RF Technology, Frontiers Science Center for Mobile Information Communication and Security, Southeast University, Nanjing 210096, China.

D.-W. Yue, K.-X. Hou, C.-H. Li

State Key Laboratory of Coordination Chemistry, School of Chemistry and Chemical Engineering, Nanjing University, Nanjing 210023, China

Email: [chli@nju.edu.cn](mailto:chli@nju.edu.cn)

B.-Y. Yu, L. Ju, Z.-G. Liu, Y.-Q. Dai, W.-B. Lu

Purple Mountain Laboratories, Nanjing 211111, China.

Y.-Q. Dai, Y.-S. Guan, Q. Li

Institute of Advanced Materials and School of Chemistry and Chemical Engineering, Southeast University, Nanjing 211189, China.

Email: [quanli3273@gmail.com](mailto:quanli3273@gmail.com)

H. K. Bisoyi, Q. Li.

Advanced Materials and Liquid Crystal Institute and Chemical Physics Interdisciplinary Program, Kent State University, Kent, OH 44242, USA.

**Fig. S1.** Synthesis of **ATPA-EP** polymer. (a) Toluene, 130 °C, 12 h, reflux. (b) 80 °C, 4 h.


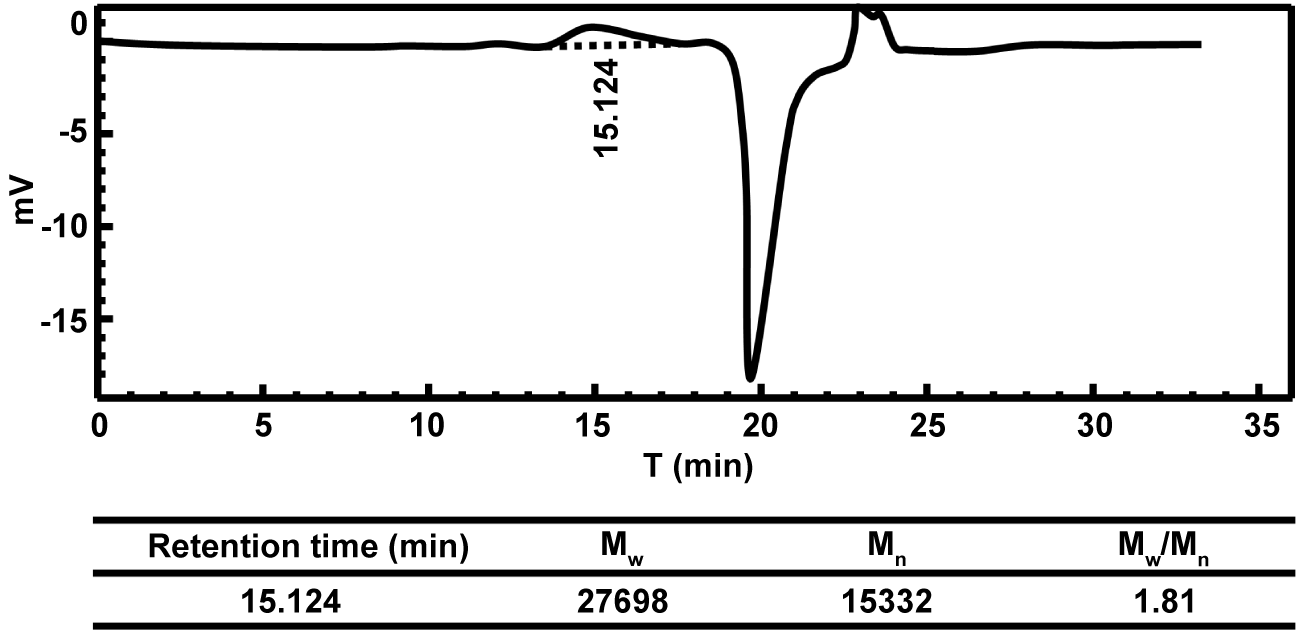


**Fig. S2.** The GPC elution figure of **ATPA-EP**.


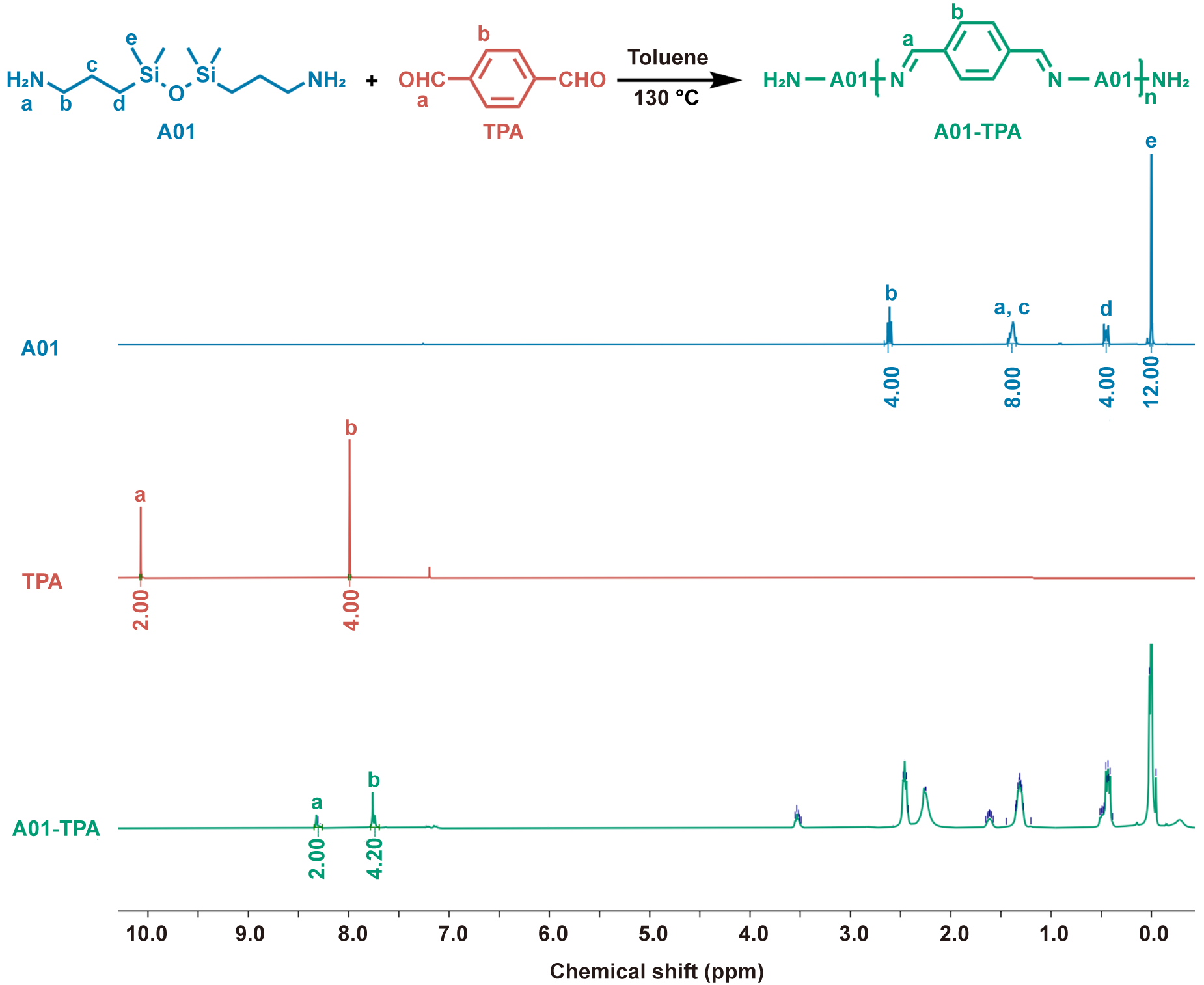


**Fig. S3.** Synthesis route and the ^1^H NMR spectra of A01, TPA, and **A01-TPA** in DMSO-*d*_6_ at 25 °C.


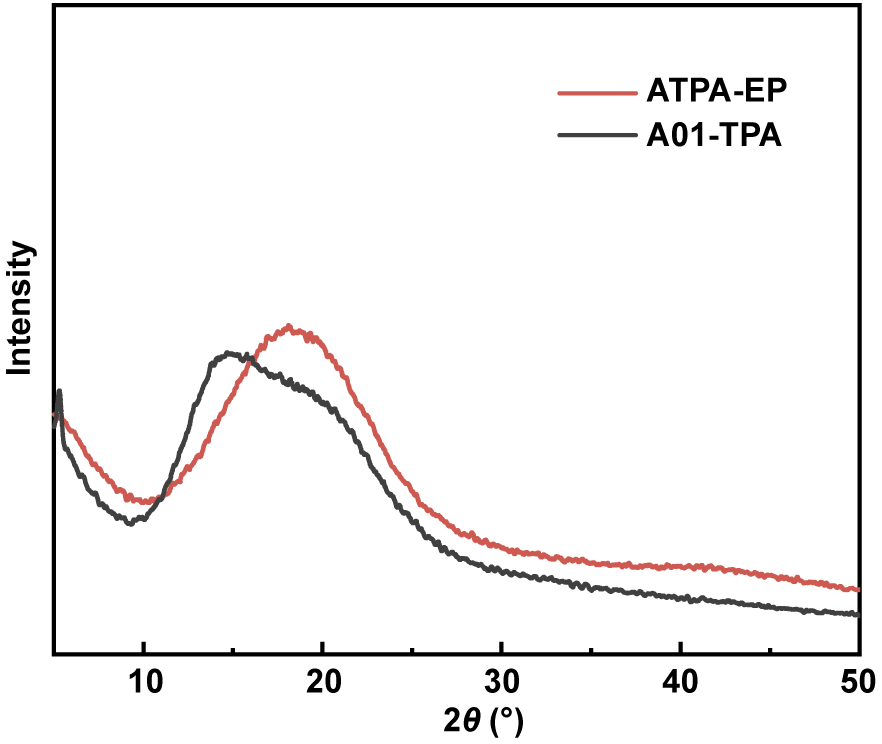


**Fig. S4.** The typically amorphous peaks in XRD patterns of **A01-TPA** and **ATPA-EP**, indicating no obvious aggregation of both products.


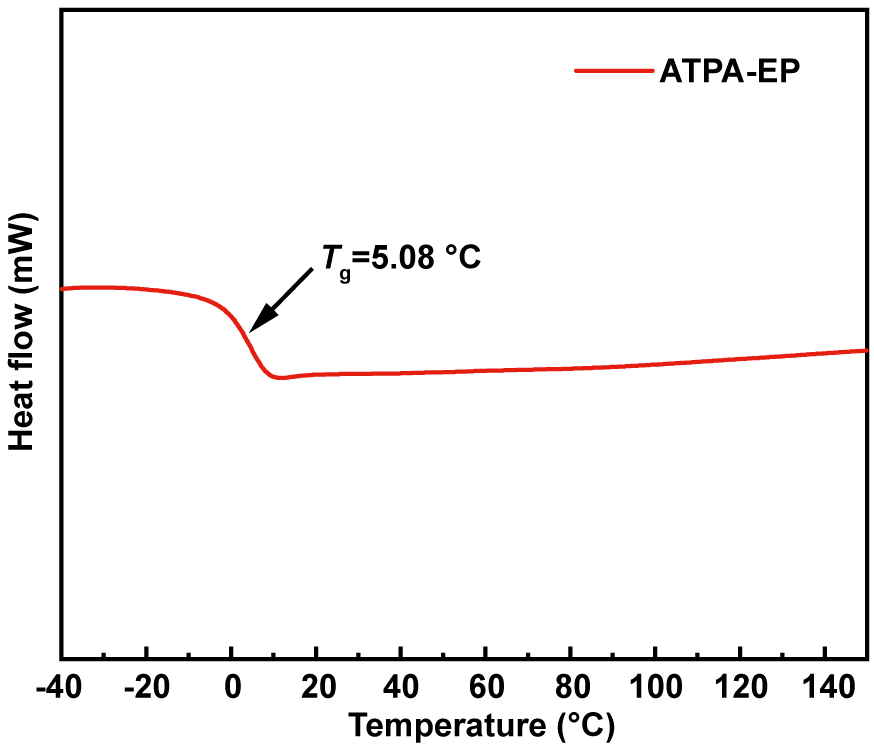


**Fig. S5.** DSC thermal grams and the glass transition point (*T*_g_) of **ATPA-EP**.


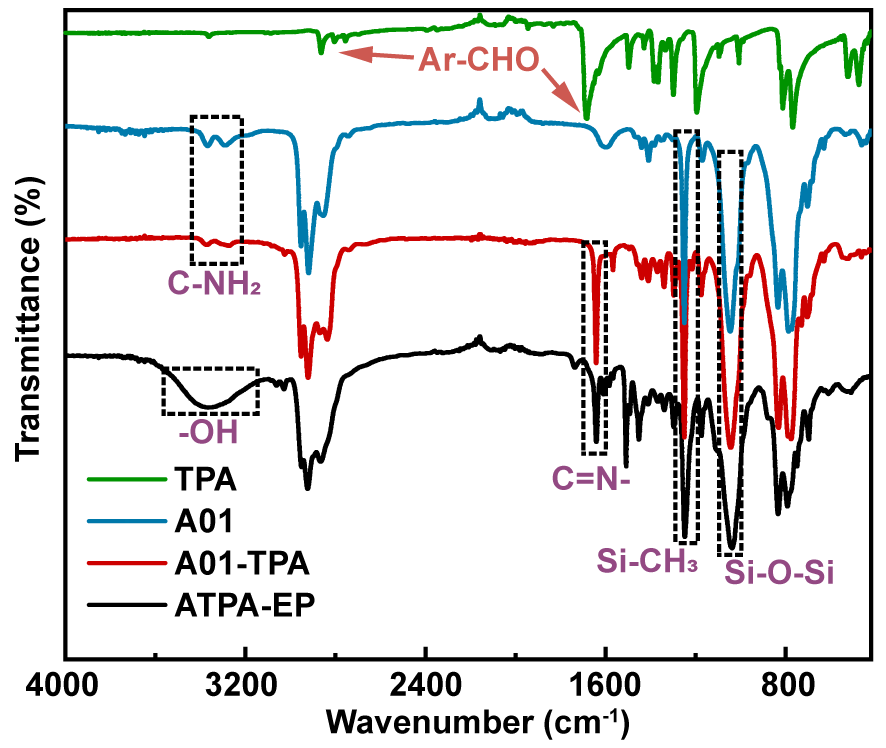


**Fig. S6.** FT-IR spectra of reactants and **A01-TPA** and **ATPA-EP**. TPA shows a unique aldehyde C=O absorption peak around 1684 cm^-1^, after reacting with A01, the C=O vibration peak totally disappeared in the resultant **A01-TPA**, and a new absorption peak of C=N appeared at 1642 cm^-1^. The broad peak of **ATPA-EP** around 3400 cm^-1^ was assigned to the hydroxyl group.


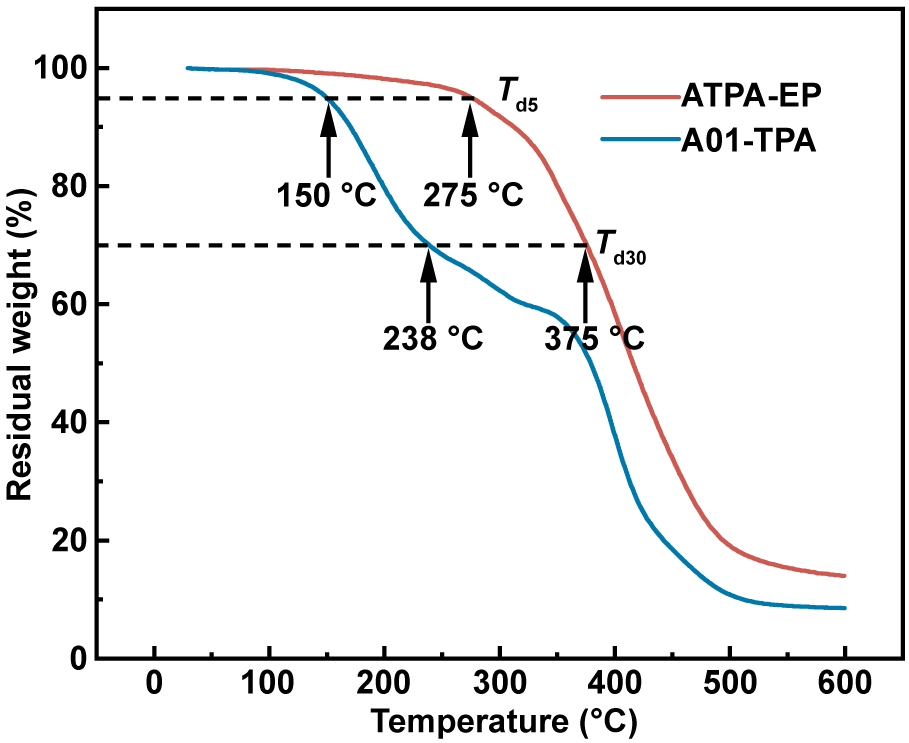


**Fig. S7.** TGA thermal grams of **A01-TPA** and **ATPA-EP**. *T*_d5_ and *T*_d30_ is defined as the 5% and 30% weight loss point respectively.


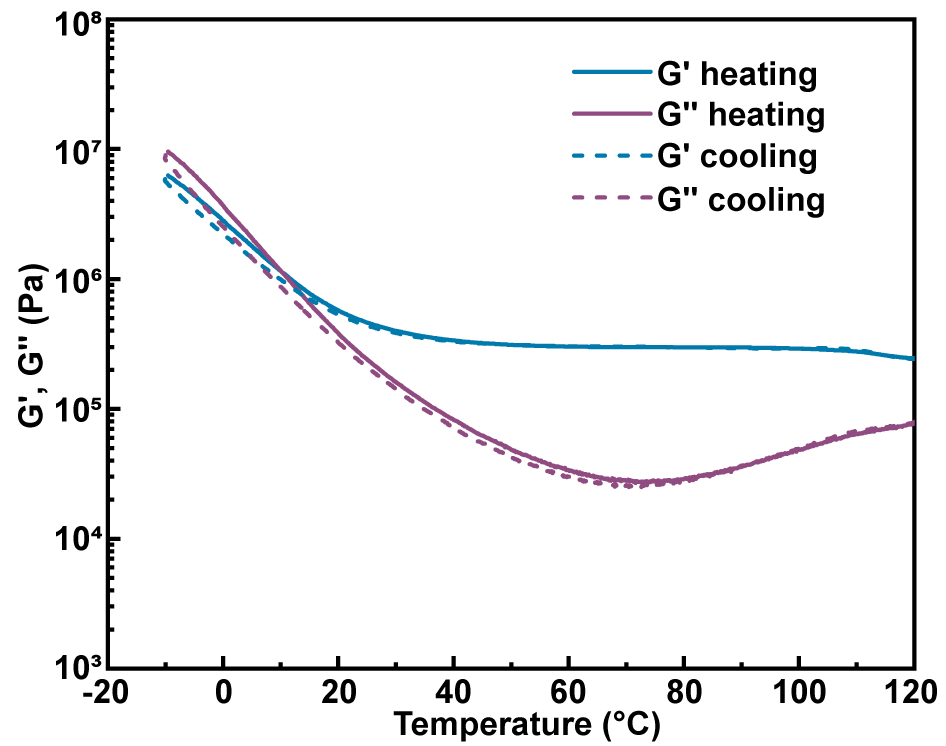


**Fig. S8.** Dynamic oscillatory temperature sweeps of **ATPA-EP**.


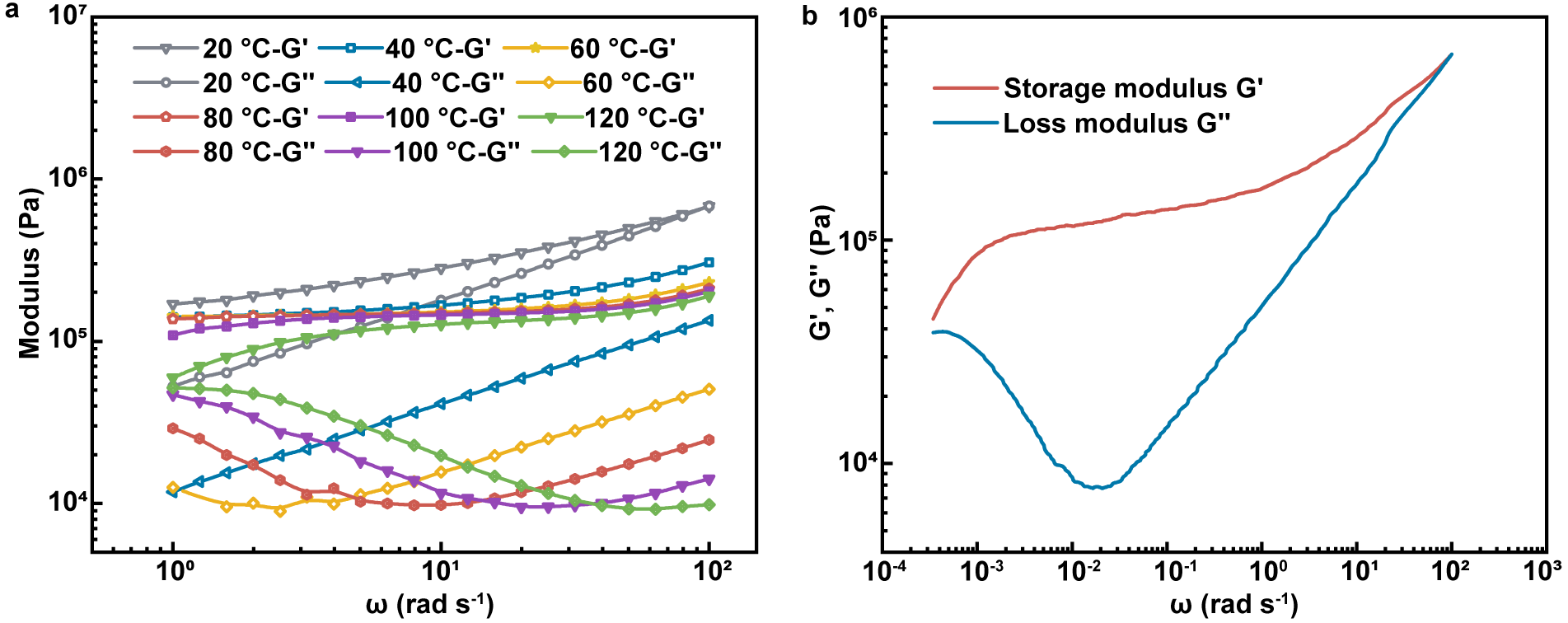


**Fig. S9.** Cooling scan SAOS data of **ATPA-EP** at reference temperature of 20 °C. (a) Cooling scan SAOS curves at selected temperature. (b) The corresponding time-temperature superposition master curve of **ATPA-EP**.


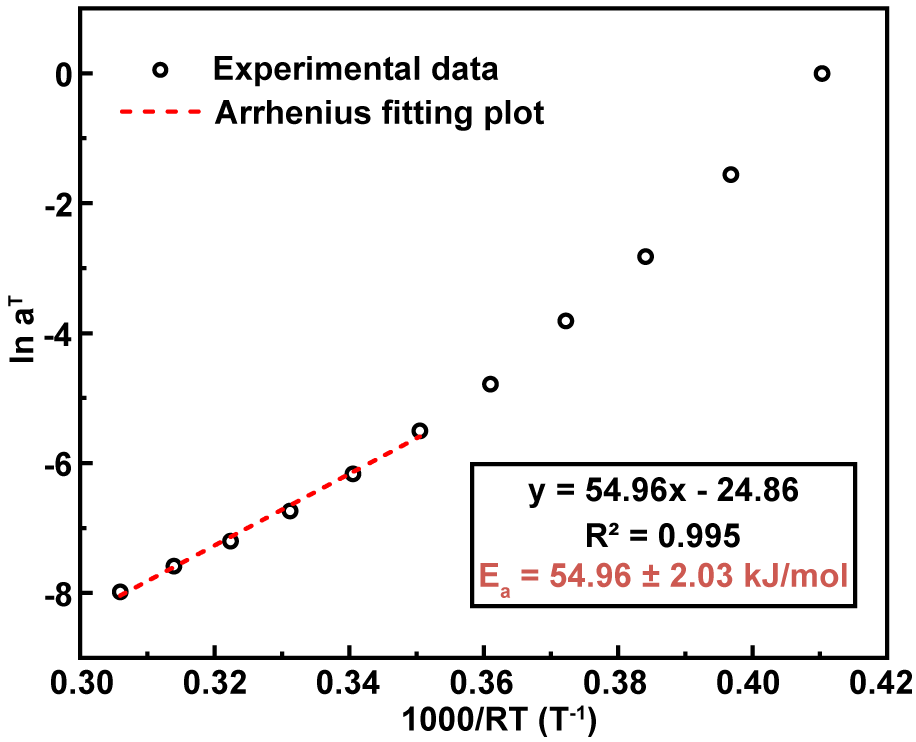


**Fig. S10.** Arrhenius fitting curve of ln a_T_ as a function of temperature.


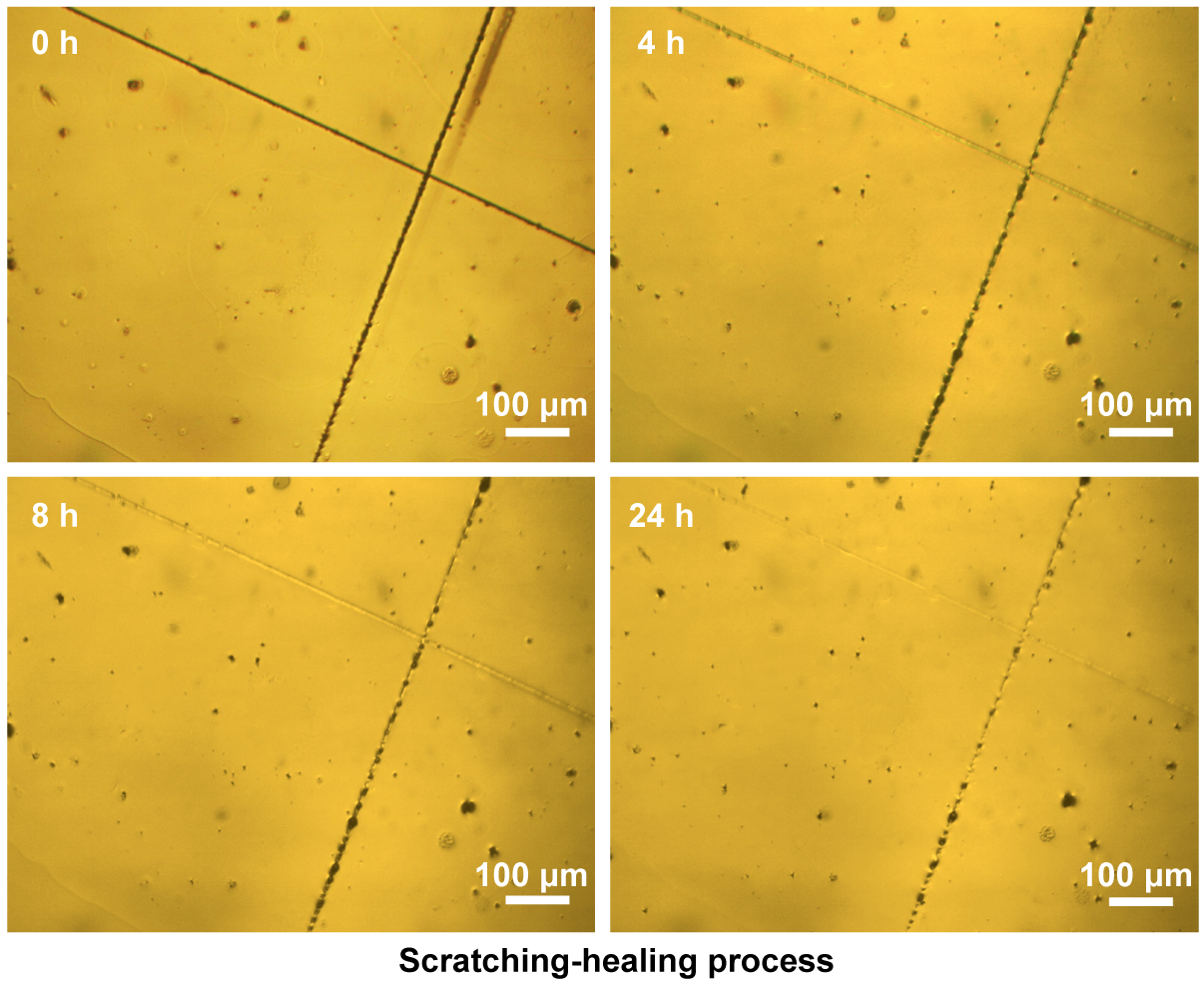


**Fig. S11.** Optical microscopy images of the scratched **ATPA-EP** film after self-healing for different time at room temperature.


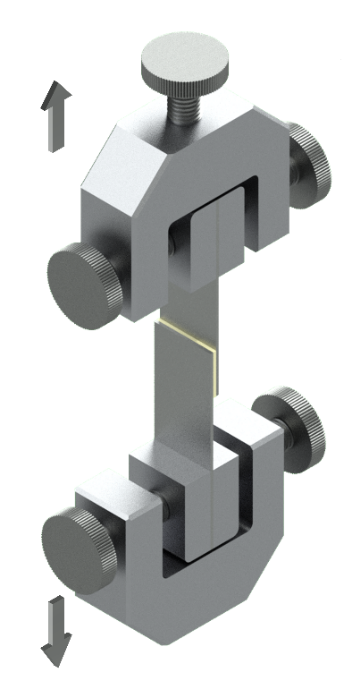


**Fig. S12.** Schematic of the lap shear strength tests.


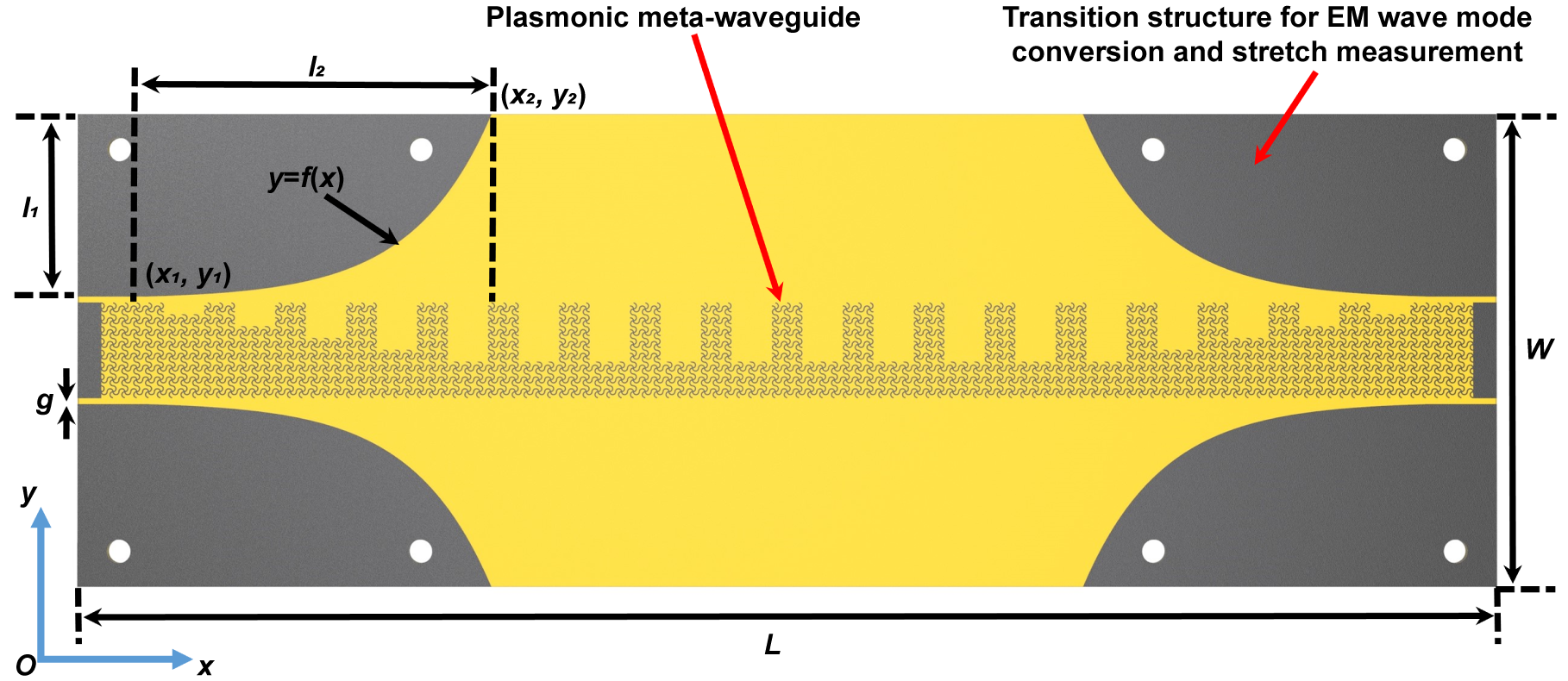


**Fig. S13.** The detailed geometric parameters of the fabricated spoof plasmonic meta-waveguide.

*L* = 120 mm, *W* = 40 mm, *l_1_* = 15.45 mm, *l_2_* = 29 mm, *g* = 0.5 mm. The boundary of the transition structure can be expressed by the following formula:

$$y\left( x \right)=C_{1}\times e^{ax}+C_{2} (x_{1}<x<x_{2})$$

$$C_{1}=\frac{y_{2}-y_{1}}{e^{ax_{2}}-e^{ax_{1}}} ,C_{2}=\frac{y_{1}\times e^{ax_{2}}-y_{2}\times e^{ax_{1}}}{e^{ax_{2}}-e^{ax_{1}}}$$

where *a* = 0.15, *x_1_* = 6 and *x_2_* = 35.


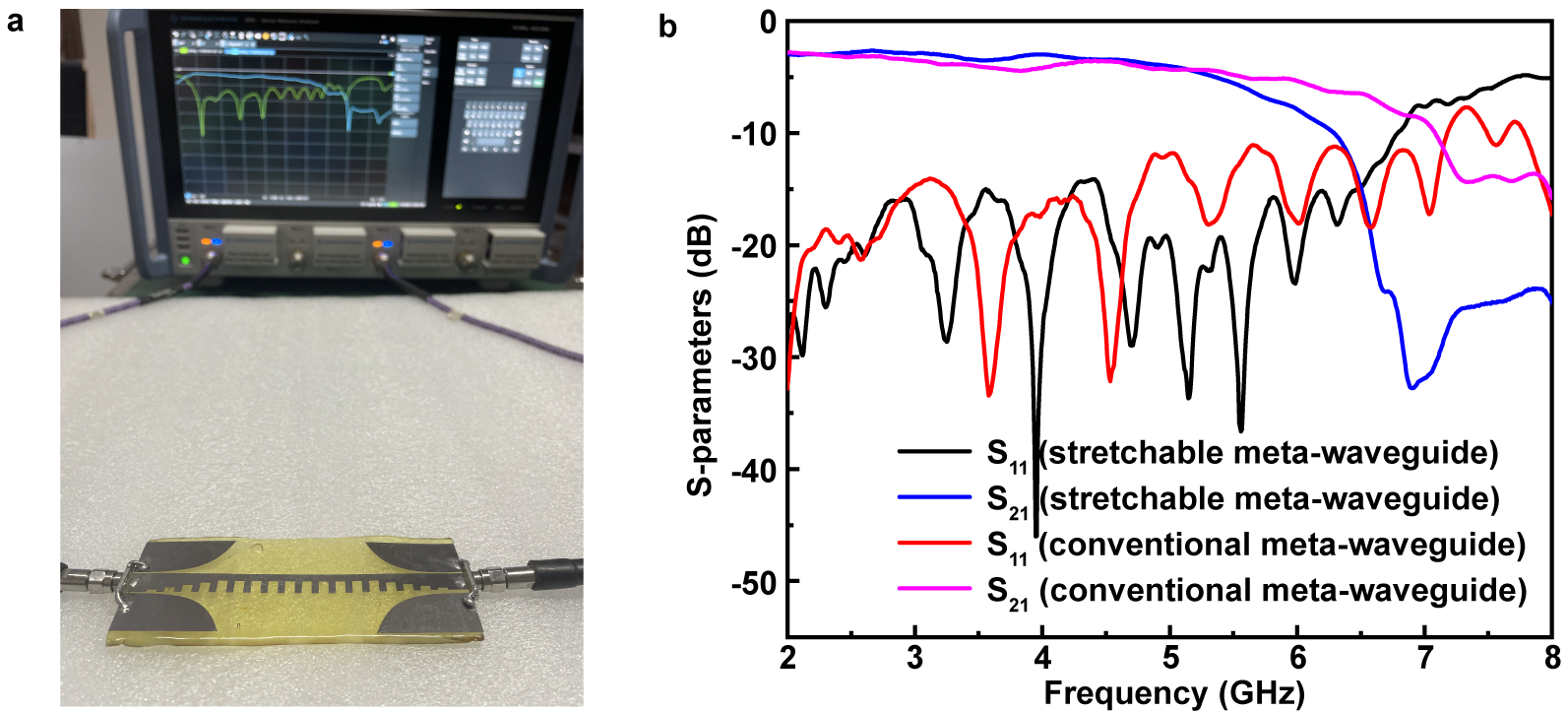


**Fig. S14**.Comparative experiment between the proposed stretchable meta-waveguide and conventional meta-waveguide. (a) Measurement photo of the meta-waveguide based on conventional metal. (b) Measured S-parameters of the stretchable meta-waveguide and conventional meta-waveguide.


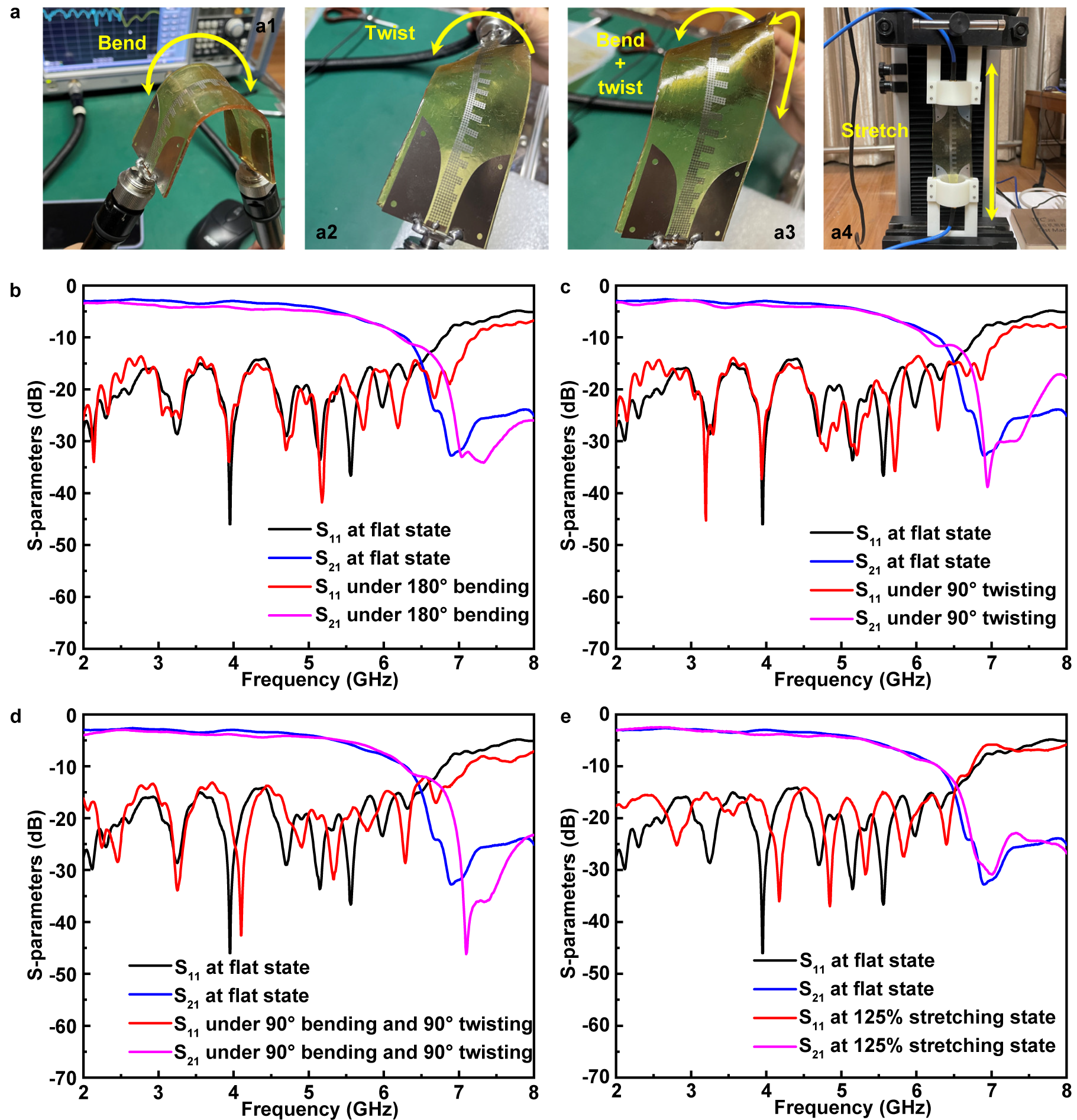


**Fig. S15.** Detailed measurement photos and results of the deformation experiment. **a** Deformation measurement photos of the spoof plasmonic meta-waveguide sample. Detailed S-parameters of the meta-waveguide under **b** 180° bending, **c** 90° twisting, **d** 90° bending and 90° twisting, and **e** 125% stretching state.


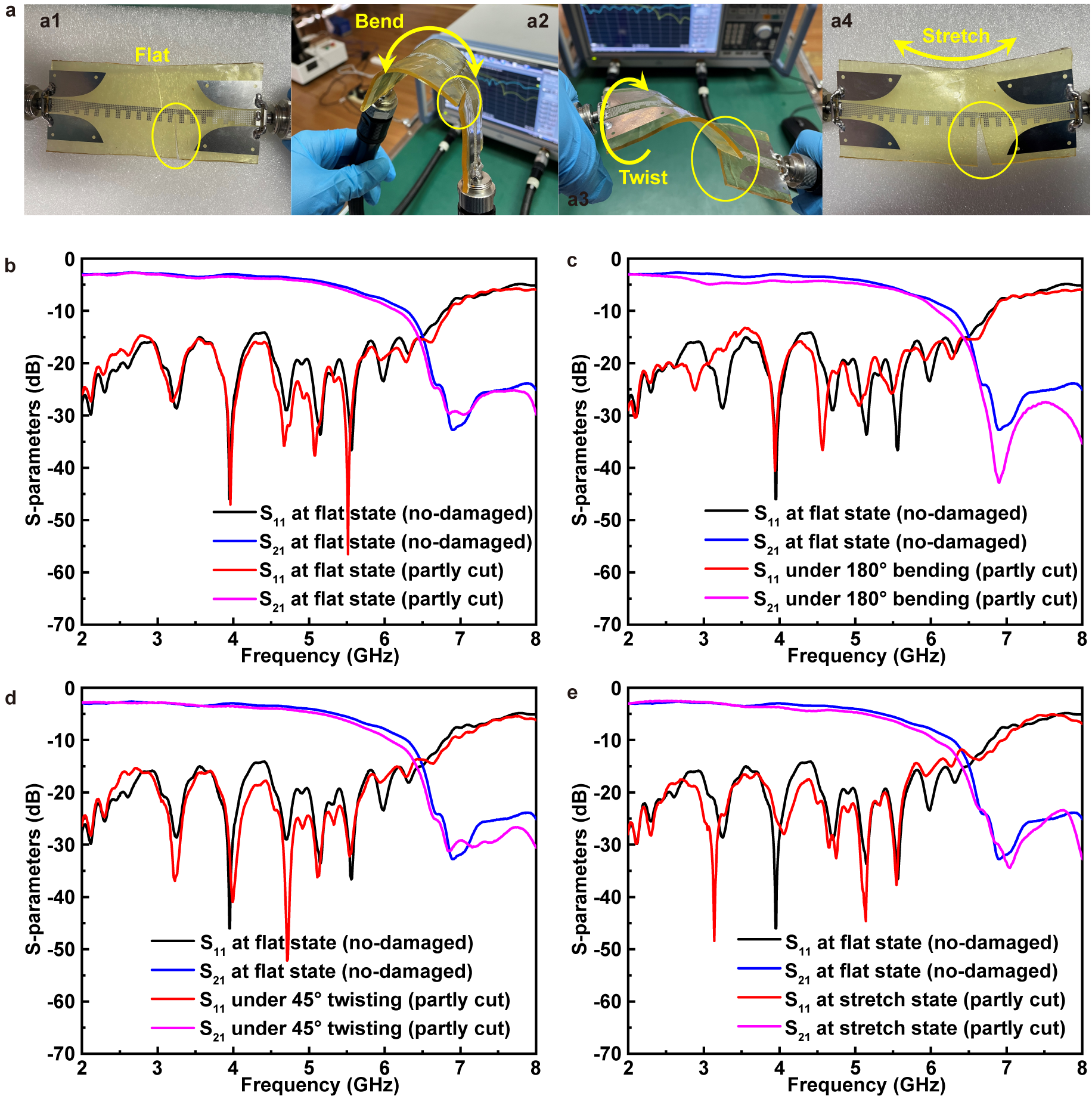


**Fig. S16.** Detailed measurement photos and results of the damage resistance experiment. **a** Damage resistance measurement photos of the meta-waveguide sample. Detailed S-parameters of the partly damaged spoof plasmonic meta-waveguide under **b** flat state, **c** 180° bending, **d** 45° twisting, and **e** stretching state.


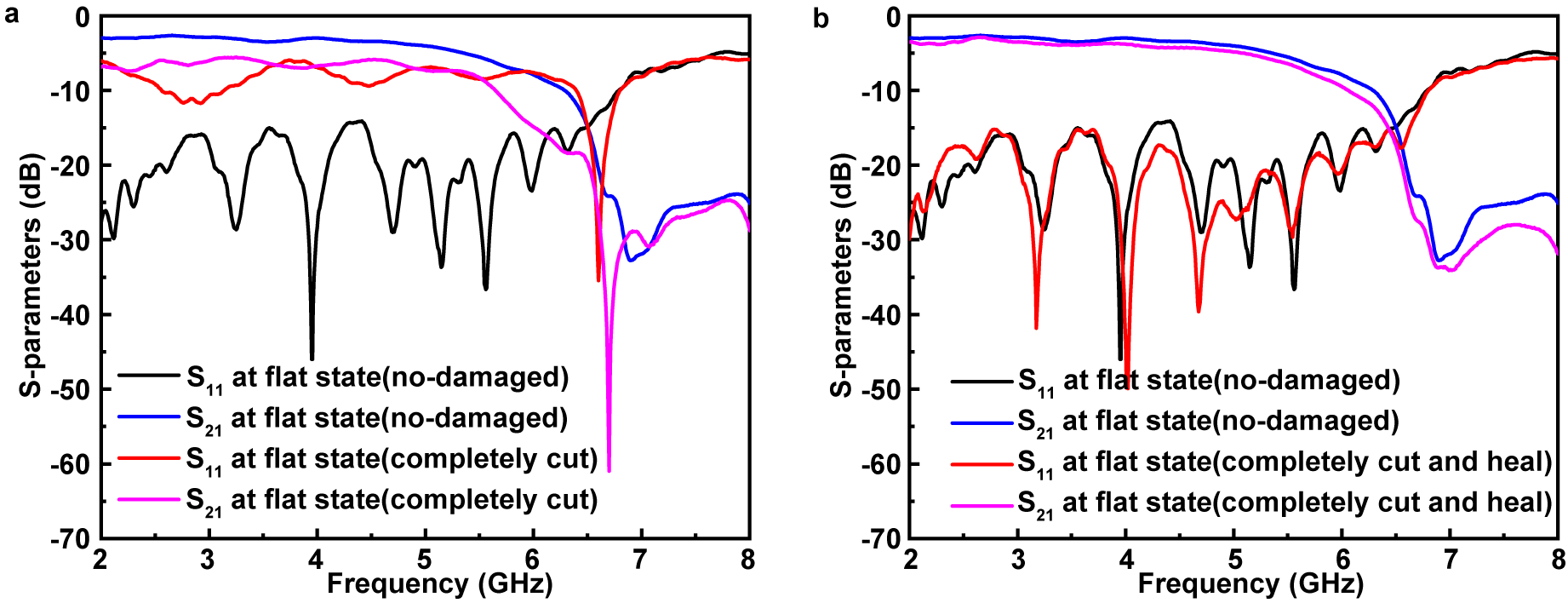


**Fig. S17.** Detailed S-parameters of the of the completely broken spoof plasmonic meta-waveguide under **a** broken state and **b** healed state.


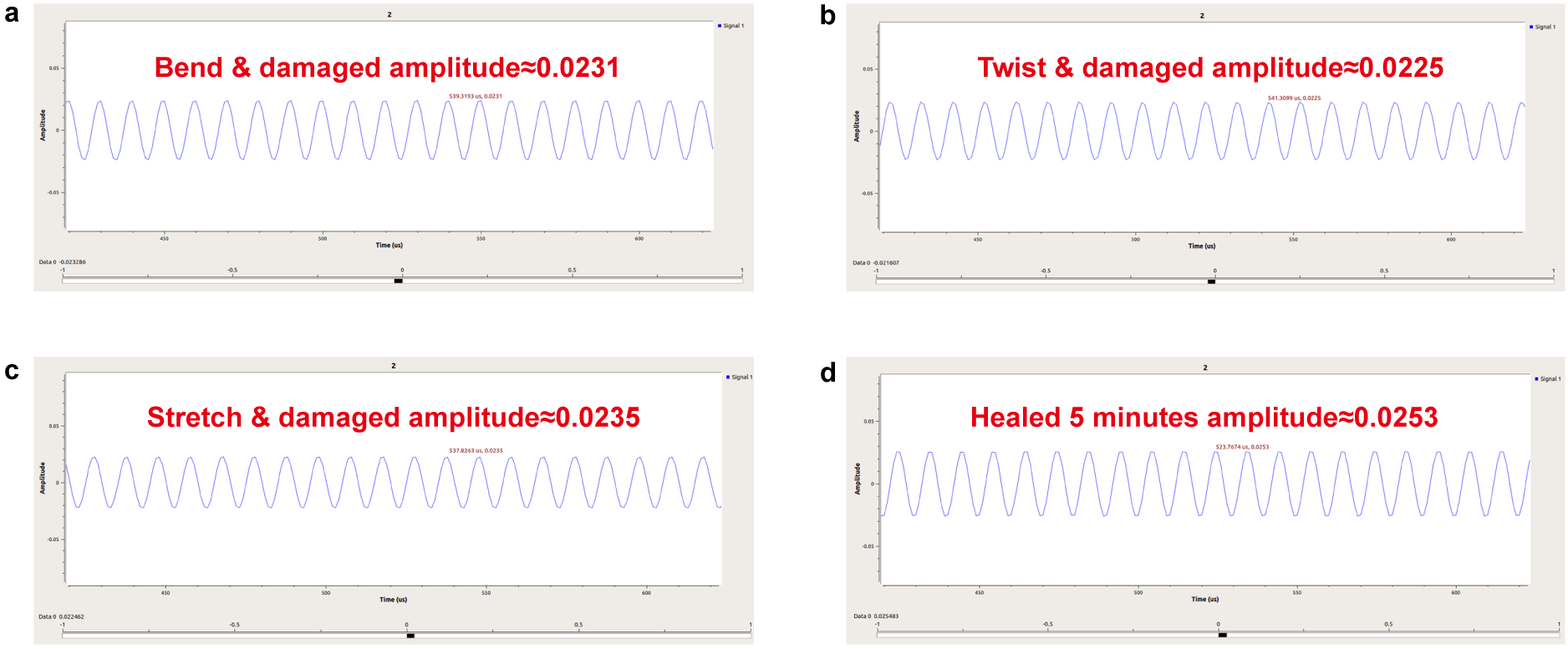


**Fig. S18.** Detailed received waveforms of the of the partly damaged spoof plasmonic meta-waveguide under **a** bend state, **b** twist state, **c** stretch state, and **d** healed state.
